# Supplementary material for: Zoonotic RVA: State of the Art and Distribution in the Animal World
Source: Viruses. 2022 Nov 18;14(11):2554. doi: 10.3390/v14112554 (PMC9694813; doi:10.3390/v14112554)
Supplement: Supplementary file 1 [file viruses-14-02554-s001.zip › viruses-2013301-supplementary.pdf]

viruses

Supplementary Materials:

Table S1. Combination of G and P genotypes.

GP[]	G1	G2	G3	G4	G5	G6	G7	G8	G9	G10	G11	G12	G13	G14	G15	G16	G17	G18	G19	G20	G21	G22	G23	G24	G25	G26	G27	G28	G29	G30	G31	G32	G33	G34	G35	G36	G37	G38	G39	G40	G41
P[1]			mky		Cow	1		2		3																															
P[2]			4																																						
P[3]			5						Pig															mky			sgl														
P[4]		Pig																																							
P[5]	Cow	Cow	Cow	6	Cow	7		Cow		8																															
P[6]	Pig	Pig	Pig	9	Pig			Pig	Pig	Pig	Pig	Pig													Bat	Pig															
P[7]	9		Pig	Pig	10	Cow		6	Pig	Pig	Pig	Pig	Pig																												
P[8]	lma		lma			Cow			pig			6																													
P[9]			11																																						
P[10]			Bat																																						
P[11]			alp			12		13		gfe	Pig				Cow																										
P[12]			hrs											hrs																											
P[13]		Pig	Pig	Pig	Pig	Pig			9		Pig															Pig															
P[14]			14			anl		15		16					Cow														Buf												
P[15]						dee				17																															
P[16]						mou										mou																									
P[17]							trk											18																rcn							
P[18]													Horse																												
P[19]			Pig	Pig					Pig										gfw																						
P[20]																mou																									
P[21]															Cow																										
P[22]			rbt	Pig																																					
P[23]		Pig	Pig	9	Pig	Pig			9																																
P[24]			mky																														Cow								

P[25]				Pig																																					
P[26]					Pig						Pig																														
P[27]	Pig	Pig	Pig	Pig	Pig						Pig																														
P[28]*																				Hmn																					
P[29]																					Cow																				
P[30]																			chk																						
P[31]																			19																						
P[32]		Pig									Pig																														
P[33]																								Cow																	
P[34]		Pig		Pig							Pig																														
P[35]							20															21																			
P[36]																											sgl														
P[37]																							phe																		
P[38]																	trk																								
P[39]																												gul													
P[40]			alp																																						
P[41]*																													Hmn												
P[42]																														Bat	Bat										
P[43]																									Bat																
P[44]**																																									
P[45]			Rat																																						
P[46]																																22									
P[47]																				Bat										Bat											
P[48]																																	Bat								
P[49]				Pig					Pig																																
P[50]																																			alp						
P[51]																																				Bat					

P[52]																																						cow					
P[53]																															Bat												
P[54]																																							Bat				
P[55]																																								shw			
P[56]																																									crw		
P[57]																																										Mou	

* These genotypes were reported only in humans.

** New genotype recognized by RCWG in strain RVA/Bat-wt/NLD/NPpipi1/2014/GxP[44].

Hosts were named as follows: alp: alpaca, atd: anatid, bat: bat, bea: bear, boa: boar, buf: buffalo, cml: camel, cat: cat, chk: chiken, clm: clam, crw: crow, cow: cow, dee: deer, dog: dog, dov: dove, gfe: giraffe, goa: goat, gnc: guanaco, gfw: guinea fowl, gul: gull, hwk: hawk, hrs: horse, lma: lama, mky: monkey, mou: mouse, phe: pheasant, pig: pig, rbt: rabbit, rcn: racoon, fox: fox, sqi: squirrel, rat: rat, anl: sable antelope, shp: sheep, shw: shrew, sgl: sugar glider, trk: turkey, sco: velvet scooter, vcu: vicugna, hmn: human.

Some cells have more than one host, and we codified it for a better lecture. 1: Cow, Turkey, Ostrich, Horse, Pig, Goat. 2: Dog, Monkey, Lama, Horse, Goat, Cow, Guanaco, Alpaca, Pig. 3: Ostrich, Horse. 4: Monkey, Bat, Hawk. 5: Cow, Cat, Dog, Monkey, Bat, Horse, Goat, Mouse, Rat. 6: Cow, Pig. 7: Horse, Cow. 8: Cow, Pig, Goat. 9: Pig, Boar. 10: Pig, Horse, Cow. 11: Cat, Racoon, Pig. 12: Rabbit, Cow. 13: Camel, Cow. 14: Rabbit, Alpaca. 15: Alpaca, Vicugna, Lama, Sheep, Guanaco, deer, Cow. 16: Goat, Cow. 17: Camel, Sheep. 18: Velvet Scooter, Red Fox, Guinea Fowl, Dove, Cow, Chicken. 19: Chicken, Guinea Fowl. 20:Chicken, Turkey. 21: Turkey, Cow. 22: Shrew, Mouse.

Table S1. In columns are the VP4 genotypes, and rows have VP7 genotypes. Intersections indicate in which species the combination was reported. Lockers with more than one specie were coded whith numbers.

Table S2. VP7 genotypes.

	G1	G2	G3	G4	G5	G6	G7	G8	G9	G10	G11	G12	G13	G14	G15	G16	G17	G18	G19	G20	G21	G22	G23	G24	G25	G26	G27	G28	G29	G30	G31	G32	G33	G34	G35	G36	G37	G38	G39	G40	G41	Total
Alpaca	4		11					4																											1							20
Anatid																																										0
Bat	1		16																	2					3					4	1		1			2		1				31
Bear	1																																									1
Buffalo	1		6			3		1		2																			1													14
Camel	1							1		2																																4
Chicken							1											1	75																							77
Clam	11	2	18						16			1																														48
Gull																												1														1
Cow	21	12	38	12	16	531		99	12	173	1	15			7		1	1			3			1													1					944
Crow																																								1		1
Deer						1		1																																		2
Dog			42					9	1	2								1																								55
Donkey																																										0
Dove																	1	24																								25
Cat			7																																							7
Giraffe										1																																1
Goat			3			15		13		5				1																												37
Guanaco								2																																		2
Guinea Fowl																			18																							18
Hawk			1																																							
Horse			295		1	1		1		1			2	239																												540
Lama	6		4					4																																		14
Monkey	1		19		1			3		1																																25

Mouse	1		1													20												1				1									1	25
Ostrich						1				1																																2
Pheasant																							5																			5
Pig	15	9	96	149	170	3		13	46	2	27	1											247			8																786
Rabbit			2			1																																				3
Racoon																																		1								1
Red Fox																		1																								1
Red Squirrel			1																																							1
Rodent			1																																							1
Sable Antelope						1																																				1
Sheep	4							2		34																																40
Shrew																												31				1							1			33
Sugar glider																											11															11
Turkey						6	1															3																				10
Velvet scooter																		1																								1
Vicuna								1																																		1
Total	67	23	561	161	188	563	2	154	75	224	28	17	2	240	7	20	2	29	93	2	3	3	252	1	3	8	11	33	2	4	1	2	1	1	1	2	1	1	1	1	1	2789

Table S2. VP7 genotypes reported for each species.

Table S3. VP7 genotypes.

	G1	G2	G3	G4	G5	G6	G7	G8	G9	G10	G11	G12	G13	G14	G15	G16	G17	G18	G19	G20	G21	G22	G23	G24	G25	G26	G27	G28	G29	G30	G31	G32	G33	G34	G35	G36	G37	G38	G39	G40	G41	Total
Alpaca	4		11					4																											1							20
Anatid																																										0
Bat	1		16																	2					3					4	1		1			2		1				31
Bear	1																																									1
Buffalo	1		6			3		1		2																			1													14
Camel	1							1		2																																4
Chicken							1											1	75																							77
Clam	11	2	18						16			1																														48
Gull																												1														1
Cow	21	12	38	12	16	531		99	12	173	1	15			7		1	1			3			1													1					944
Crow																																								1		1
Deer						1		1																																		2
Dog			42					9	1	2								1																								55
Donkey																																										0
Dove																	1	24																								25
Cat			7																																							7
Giraffe										1																																1
Goat			3			15		13		5				1																												37
Guanaco								2																																		2
Guinea Fowl																			18																							18
Hawk			1																																							
Horse			295		1	1		1		1			2	239																												540
Lama	6		4					4																																		14

Monkey	1		19		1			3		1																															25
Mouse	1		1													20												1				1									1	25
Ostrich						1				1																																2
Pheasant																							5																			5
Pig	15	9	96	149	170	3		13	46	2	27	1											247			8																786
Rabbit			2			1																																				3
Racoon																																		1								1
Red Fox																		1																								1
Red Squirrel			1																																							1
Rodent			1																																							1
Sable Antelope						1																																				1
Sheep	4							2		34																																40
Shrew																												31				1							1			33
Sugar glider																											11															11
Turkey						6	1															3																				10
Velvet scooter																		1																								1
Vicuna								1																																		1
Total	67	23	561	161	188	563	2	154	75	224	28	17	2	240	7	20	2	29	93	2	3	3	252	1	3	8	11	33	2	4	1	2	1	1	1	2	1	1	1	1	1	2789

Table S3. VP7 genotypes reported for each species.

Table S4. VP6 genotypes.

	I1	I2	I3	I4	I5	I6	I7	I8	I9	I10	I11	I12	I13	I14	I15	I16	I17	I18	I19	I20	I21	I22	I23	I24	I25	I26	I27	I28	I29	I30	I31	Total
Alpaca	2	4						11					2																			19
Anatid				1																												1
Bat		1	4					3					2		2							9	1		1					10		33
Bear					1																											1
Buffalo		2																														2
Camel		8																														8
Cat		1	6																													7
Chicken				11							69																					80
Clam																																0
Gull																					1											1
Cow	1	147		1	51																											200
Crow																										1						1
Deer		3																														3
Dog	1	11	6		1																											19
Donkey																																0
Dove				58							1										1											60
Giraffe		1																														1
Goat		8								1																						9
Guanaco		2																														2
Guinea fowl											9																					9
Hawk																																0

Horse		35	1		1	44																										81
Lama	4	3						2																								9
Monkey		15			1				2																							18
Mouse	1						1																	1							1	4
Ostrich																																0
Pheasant				2																												2
Pig	30	5			234							1		2																		272
Rabbit		3															1															4
Racoon																										1						1
Red Fox				1																												1
Red Squirrel																																0
Rodent																				1												1
Sable Antelope		1																														1
Sheep		5								2																						7
Shrew																								1			1					2
Sugar glider																			1													1
Turkey				5																												5
Velvet scooter				1																												1
Vicuna		1																														1
Total	39	256	17	80	289	44	1	16	2	3	79	1	4	2	2	0	1	0	1	1	2	9	1	2	1	2	1	0	0	10	1	867

Table S4. VP6 genotypes reported for each species.

Table S4. Partial Pigs constellations.

Host	Strain	G	P[]	I	R	C	M	A	N	T	E	H	Country	Reference/ Acc. number
Pig	MRC-DPRU1513	5	6	5	1	1	1	8	1	1/7	9	1	South Africa	KP753000-KP753011
	MRC-DPRU1567	5	6	5	1	1	1	8	1	1/7	1	1	South Africa	KJ752475-KJ752491
	MRC-DPRU1568	5	x	5	1	1	1	8	1	1	9	1	South Africa	KP753185-KP753195
	LS00006_OSU	5	x	5	1	1	1	1	1	1	1	1	USA	KR052752-KR052761
	MRC-DPRU1562	5	x	5	1	1	1	8	1	7	1	1	South Africa	KP752851-KP752860
	MRC-DPRU1576	5	x	5	1	1	1	8	1	1/7	1/9	1	South Africa	KP752759-KP752773
	MRC-DPRU1522	5/9	x	5	1	1	1	8	1	1/7	1	1	South Africa	KP752916-KP752928
	MRC-DPRU1487	5/3	23	5	1	1	1	8	1	1	1	1	South Africa	KP753115-KP753128
	MRC-DPRU1557	4/5	23	5	1	1	1	8	1	1/7	1	1	South Africa	KP752951-KP752965
	MRC-DPRU1540	6/9	x	1	x	x	x	x	1	1	1	1	South Africa	KJ753680-KJ753686
	AB82	2	34	5/14	1	1	1	8	1	7	9	1	Canada	[152]
	SUI13A	5	13	5	1	1	x	8	x	7	1	1	Brazil	MH785306-MH785339-MH785323-MH785370-MH785353-MH785460-MH785443-MH785427-MH785398
	MRC-DPRU3878	5	x	2/5	x	1	x	8	1	1/7	1	1	South Africa	KP753056-KP753065
	sh0902	1	7	x	x	x	x	x	x	x	1	x	Czech Republic; China	[85]
	P21-5	1	27	x	x	x	x	x	x	x	9	x	Slovenia	[153]
	103-P-003-2-0443	2	13	5	x	x	x	x	x	x	x	x	Taiwan	[135]

	CE-M-06-0003	2	27	14	x	x	x	x	x	x	x	x	Canada	[154]
	CMP034	2	27	5	x	x	x	x	x	x	9	1	Thailand	[155]
	103-P-004-1-0575	3	19	5	x	x	x	x	x	x	x	x	Taiwan	[135]
	SCMY	3	13	5	x	x	x	x	x	x	x	x	China; Taiwan	MH320795-MH320797
	103-P-003-1-0429	3	13	12	x	x	x	x	x	x	x	x	Taiwan	[135]
	P211	4	6	5	x	x	x	x	x	x	x	x	Czech Republic	[156]
	CE-M-05-0085	4	6	5	x	x	x	x	x	x	1	x	Canada	[154]
	CE-M-05-0091	4	6	5	x	x	x	x	x	x	9	x	Canada	[154]
	103-P-004-1-0217	4	13	5	x	x	x	x	x	x	x	x	Taiwan	[135]
	CE-M-06-0010	4	13	5	x	x	x	x	x	x	1	x	Canada	[154]
	103-P-003-1-1022	4	19	12	x	x	x	x	x	x	x	x	Taiwan	[135]
	P70	4	25	1	x	x	x	x	x	x	x	x	Czech Republic	[156]
	103-P-001-1-1323	5	23	5	x	x	x	x	x	x	x	x	Taiwan	[135]
	CE-M-05-0081	5	27	5	x	x	x	x	x	x	1	x	Canada	[154]
	CE-M-06-0005	5	6	5	x	x	x	x	x	x	1	x	Canada	[154]
	P245	5	13	5	x	x	x	x	x	x	x	x	Czech Republic; Taiwan	[156]
	HP140	6	13	2	x	x	x	x	x	x	1	1	India	[157]
	103-P-002-1-0514	9	13	12	x	x	x	x	x	x	x	x	Taiwan	[135]
	103-P-001-1-0127	9	13	5	x	x	x	x	x	x	x	x	Taiwan	[135]
	LLP48	9	6	1	x	x	x	x	1	x	1	x	China	KJ126815-KJ126825-KJ126820-KJ126830-KJ126835
	CE-M-05-0067	9	6	5	x	x	x	x	x	x	1	x	Canada	[154]
	1-4	9	19	5	x	x	x	x	1	1	1	1	Taiwan	[135]
	P828	9	23	5	x	x	x	x	x	x	1	x	Czech Republic; Taiwan	[156]

	CE-M-06-0007	11	13	5	x	x	x	x	x	x	9	x	Canada; Czech Re-public	[154]
	MRC-DPRU3825	5	x	x	x	x	x	8	1	1/7	1	1	South Africa	KJ753135-KJ753141

Table S4. Partial Pig strains´ constellations reported for each hosts. Constellation´s genes are coded as G: VP7; P[]: VP4; I: VP6 ;R: VP1;C: VP2;M: VP3;A: NSP4;N: NSP1;T: NSP2;E: NSP3; H: NSP5. Country where it was reported. Reference or NCBI accession number. Colors were arbitrarily defined from 1 - 14, and others are in white.
